# Dynamic performance and scenario-based screening strategy of six COPD questionnaires: a cross-sectional study with prevalence-driven robustness validation

**DOI:** 10.3389/fmed.2025.1666703

**Published:** 2025-09-24

**Authors:** Qinqin Wang, Lingjun Liu, Qiao Zhang, Hong Li, Qianli Ma

**Affiliations:** ^1^Department of Chronic Respiratory Disease Management and Rehabilitation Center, Chongqing Songshan General Hospital, Chongqing, China; ^2^Department of Allergy and Immunology, Chongqing Songshan General Hospital, Chongqing, China; ^3^Department of Respiratory and Critical Care Medicine, Chongqing Songshan General Hospital, Chongqing, China

**Keywords:** COPD screening, diagnostic accuracy, questionnaire comparison, prevalence effect, adaptive screening

## Abstract

**Introduction:**

Chronic Obstructive Pulmonary Disease (COPD) imposes a high global burden. Spirometry is the diagnostic gold standard but has accessibility barriers. Screening questionnaires provide a feasible alternative.

**Objectives:**

To compare the diagnostic performance and robustness of six COPD screening questionnaires (LFQ: Lung Function Questionnaire; IPAG: International Primary Care Airways Group Questionnaire; Modified-IPAG; COPD-PS: COPD Population Screener Questionnaire; COPD-SQ: COPD Screening Questionnaire; SCSQ: The Salzburg COPD Screening Questionnaire) within a single cohort population, thereby providing evidence to support targeted screening for COPD.

**Methods:**

This cross-sectional study enrolled adults ≥40 years without prior asthma or non-COPD chronic lung diseases. Participants completed six screening questionnaires and spirometry. COPD was confirmed by pulmonologists. Receiver operating characteristic (ROC) curves were constructed for each questionnaire; sensitivity, specificity, accuracy (ACC), positive predictive value (PPV), negative predictive value (NPV), and area under the curve (AUC) were calculated. Dynamic variations in screening performance were simulated under different disease prevalence scenarios.

**Results:**

Modified-IPAG and LFQ showed highest sensitivity (94.78%/91.79%) and NPV (98.11%/97.45%); COPD-PS and COPD-SQ had highest specificity (79.32%/87.05%) and PPV (43.50%/43.87%). AUC ranged 0.681 (SCSQ)–0.796 (COPD-PS). Dynamic simulations revealed COPD-PS maintained stable ACC across prevalence (ΔACC = 0.06; β = −0.018; *P* = 0.114), while SQ declined with increasing prevalence (ΔACC = 0.26; β = −0.263; *P* < 0.001).

**Conclusion:**

A “Scenario-Priority” strategy is proposed: For rule-out screening, use high-sensitivity tools (Modified-IPAG/LFQ); for high-risk identification, prioritize robust COPD-PS; in low-prevalence regions (<30%), use high-specificity SQ. This approach transcends the conventional “tool-first” static framework, delivering evidence-based support for precision COPD screening implementation.

## 1 Introduction

Chronic obstructive pulmonary disease (COPD) is a chronic respiratory disorder characterized by persistent airflow limitation, imposing a substantial global disease burden (1, 2). As the third leading cause of death worldwide, COPD disproportionately affects low- and middle-income countries, accounting for 90% of premature COPD-related deaths (3). The 2019 global prevalence reached 10.3% (approximately 391.9 million cases) in adults aged 30–79 years (4), while Chinese data report higher rates (13.7% in adults ≥40 years) with significant regional disparities (5). As the disease progresses, symptoms such as dyspnea and restricted activity frequently lead to high disability-adjusted life years and diminished quality of life among COPD patients (6–8).

Research indicates that early COPD screening enables smoking cessation interventions to delay lung function decline and reduces hospitalization costs associated with acute exacerbations (9–11), demonstrating significant health economic value. Although spirometry remains the diagnostic gold standard for COPD (1), its utility as a population-wide screening tool is debated (12–14). In resource-constrained primary care settings, spirometry faces substantial implementation barriers, including limited accessibility, high technical requirements, and potential suboptimal patient cooperation, resulting in underutilization of high-quality testing and diminishing its efficiency as a case-finding instrument (15–17). In contrast, brief, user-friendly self-administered questionnaires offer a pragmatic alternative for identifying high-risk individuals requiring spirometry referral, leveraging advantages such as low cost, ease of operation, and capacity for dynamic symptom monitoring (e.g., dyspnea grading).

Although multiple studies have reported the diagnostic performance of COPD screening questionnaires, significant variability exists across different investigations (18–22). This heterogeneity complicates end-users’ ability to determine which questionnaire optimally achieves the highest screening efficacy within specific target populations and clinical contexts. A key contributor to this dilemma is the pronounced dependence of questionnaire performance–particularly PPV and NPV–on the underlying disease prevalence of the target population. Previous evaluations predominantly assessed performance under single, fixed prevalence conditions, neglecting to examine diagnostic robustness across varying prevalence rates. Consequently, their findings demonstrate limited generalizability to real-world settings with divergent prevalence profiles, restricting the broader applicability of screening strategies across diverse implementation scenarios. Therefore, a systematic evaluation of COPD questionnaire performance within a unified cohort–accounting for different usage scenarios and prevalence conditions–is imperative to generate evidence-based support for precision-based screening strategies.

## 2 Materials and methods

### 2.1 Study design

A cross-sectional study was conducted among subjects referred for pre- and post-bronchodilator spirometry at the Respiratory Outpatient Clinic of Chongqing Songshan General Hospital between March 2021 and January 2023.

### 2.2 Participants

#### 2.2.1 Inclusion criteria

Aged ≥40 years; Voluntarily provided written informed consent; Completed six COPD screening questionnaires; Underwent post-bronchodilator spirometry.

#### 2.2.2 Exclusion criteria

Current acute exacerbation of respiratory disease; Inability to perform spirometry according to American Thoracic Society and the European Respiratory Society (ATS/ERS) technical standards or contraindications to spirometry; Cognitive or linguistic barriers precluding questionnaire completion.

### 2.3 Data collection and questionnaires

Prior to spirometry testing, a trained coordinator administered an integrated questionnaire to participants. This instrument consolidated items from six established COPD screening tools: the LFQ, IPAG, Modified-IPAG, COPD-PS, COPD-SQ and SCSQ. Data collection encompassed three domains: (1) demographic characteristics including age, gender, height, and weight; (2) risk factors such as history of biomass fuel exposure, long-term exposure to dust or chemical particulates, allergy history, family history of respiratory diseases, and childhood chronic respiratory disease history; and (3) respiratory symptoms comprising cough, sputum production, dyspnea, and quality-of-life impacts attributable to respiratory problems. Body mass index (BMI) was derived from measured height and weight using standard formulae. Smoking exposure was quantified as pack-years, calculated by multiplying the number of cigarette packs smoked daily (standardized at 20 cigarettes per pack) by total years of smoking.

#### 2.3.1 Questionnaires

##### 2.3.1.1 Lung function questionnaire (LFQ)

Developed by Yawn et al. (23), this 5-item tool (age, smoking, wheezing, dyspnea, sputum) initially used binary responses (yes/no), with ≥3 positive items indicating airflow limitation risk (AUC = 0.720; sensitivity 73.2%, specificity 58.2%). The validation study Hanania et al. (24) demonstrated that a 5-point scale (score 5–25) with ≤18 as the threshold achieved sensitivity 82.6%, specificity 47.8%, and ACC 54.3% (AUC = 0.652), significantly outperforming the binary format.

##### 2.3.1.2 COPD population screener questionnaire (COPD-PS)

Developed by Martinez et al. (25), including 5 items: age, smoking history, breathlessness, productive cough, and activity limitation due to breathing problems. When using a cutoff score of 5 on the COPD-PS, the sensitivity for identifying fixed airflow obstruction (AO) is 84.4%, specificity is 60.7%, PPV is 56.8%, NPV is 86.4%, and the AUC is 0.73.

##### 2.3.1.3 International primary care airways group questionnaire (IPAG)

Its core items originate from the case-finding version of the COPD Diagnostic Questionnaire (CDQ) developed by Price et al. (26, 27) for smokers aged ≥40 years. It comprises eight items: age, BMI, pack-years of smoking, weather-related cough, phlegm without a cold, morning phlegm, wheezing frequency, and allergy history (termed the IPAG 8-item questionnaire). The IPAG Working Group recommended ≥17 as the screening threshold in its operational manual (2009) (28).

##### 2.3.1.4 Modified-IPAG

In 2016, Zhang Q et al. (29) systematically revised the IPAG questionnaire for the Chinese population by removing two low-discriminatory items (“coughing up phlegm without a cold” and “morning phlegm”), adjusting BMI scoring to Chinese standards (<24, 24–28, ≥28), and adding five China-specific risk items: secondhand smoke exposure, coughing without a cold, shortness of breath, long-term dust/chemical exposure, and childhood chronic respiratory disease history (termed the IPAG 11-item questionnaire). The optimal screening threshold (≥17), determined by the ROC curve’s inflection point, achieved 82.5% sensitivity and 72.9% specificity, significantly improving screening efficacy in the Chinese cohort.

##### 2.3.1.5 COPD screening questionnaire (COPD-SQ)

COPD Screening Questionnaire Developed by Zhou YM et al. (30). Based on China’s 2002 national COPD epidemiological survey data, this instrument comprises seven items: age, smoking pack-years, BMI, cough, dyspnea, family history of respiratory diseases, and biomass fuel exposure. A screening threshold of ≥16 is recommended (sensitivity 60.6%, specificity 85.2%, ACC 82.7%).

##### 2.3.1.6 The salzburg COPD screening questionnaire (SCSQ)

The Salzburg COPD Screening Questionnaire Developed by Austrian researchers Weiss et al. (31) based on the Burden of Obstructive Lung Diseases (BOLD) study cohort in Salzburg, comprises five items: smoking history (current/former/never), breathing problems severely limiting daily activities, health restrictions when climbing multiple flights of stairs, wheezing or whistling in the chest within the past 12 months, and coughing without a cold. A threshold of ≥2 is recommended (sensitivity 69.1%, specificity 60.0%, PPV 23.2%).

#### 2.3.2 Spirometry

All participants underwent spirometry testing using the MasterScreen Pneumo pulmonary function testing system (Jaeger, Germany).

The procedures strictly adhered to quality control standards established by the ATS/ERS (32, 33). Subjects demonstrating a pre-bronchodilator forced expiratory volume in 1 s (FEV_1_) to forced vital capacity (FVC) ratio < 0.7 received 400 μg salbutamol sulfate via metered-dose inhaler with spacer chamber, with post-bronchodilator spirometry repeated 15–20 min after administration. Complete pre- and post-bronchodilator pulmonary function data were documented for all subjects.

According to the Global Initiative for Chronic Obstructive Lung Disease (GOLD) criteria (1), a post-bronchodilator FEV_1_/FVC ratio < 0.7 indicates persistent airflow limitation. After exclusion of other diseases that may cause airflow limitation, this finding supports a clinical diagnosis of COPD.

### 2.4 Statistical analysis

Descriptive statistics were employed to evaluate demographic characteristics and questionnaire scores. Continuous parametric variables were expressed as mean ± standard deviation (SD), with between-group comparisons analyzed using independent samples *t*-tests. Categorical variables were presented as frequencies and percentages (%), with between-group comparisons assessed by chi-square tests. Using GOLD criteria as the diagnostic gold standard for COPD, ROC curves were constructed for each questionnaire. Sensitivity, specificity, ACC, PPV, NPV, and AUC were calculated for all screening instruments. Differences in ROC-AUC between questionnaires were analyzed using DeLong’s test with Bonferroni correction for multiple comparisons. Linear regression analysis examined the correlation between screening ACC and disease prevalence. All statistical analyses were performed using R version 4.4.1 and MedCalc version 20.0. Statistical significance was set at *p* < 0.05.

## 3 Results

### 3.1 Participants characteristics

Among 811 initially recruited patients, 806 met the inclusion criteria and were taken to the final analysis. COPD prevalence was 16.63% (134/806). The mean age of the participants was 58.8 years (SD = 10.8), and 43.3% (349/806) were female. A diagnosis of COPD was significantly associated with male gender (83.6% vs. 16.5%, *p* < 0.001), older (66.1 ± 8.3 vs. 57.3 ± 10.7 years, *p* < 0.001), lower BMI (23.3 ± 3.4 vs. 24.1 ± 3.2, *p* = 0.007), high smoking pack-years (36.4 ± 30.2 vs. 11.6 ± 21.2, *p* < 0.001), biomass fuel exposure (25.4% vs. 12.5%, *p* < 0.001), long-term dust/chemical particle exposure (40.3% vs. 19.0%, *p* < 0.001), and a history of childhood chronic respiratory diseases (21.6% vs. 11.0%, *p* < 0.001).

By contrast, no significant association was observed with family history of respiratory diseases or allergy history (*p* > 0.05).

All six questionnaires showed statistically significant score differences between groups (*p* < 0.001). Complete data are shown in Table 1 (all *P* < 0.001).

**TABLE 1:** General characteristics of the participants.

Characteristics	Total (*N* = 806)	Non-COPD (*n* = 672)	COPD (*n* = 134)	*P-*value
**Gender, *n* (%)**				
Males	457 (56.7%)	345 (51.3%)	112 (83.6%)	<0.001*
Females	349 (43.3%)	327 (48.7%)	22 (16.5%)	
Age (years), mean (SD)	58.8 (10.8)	57.3 (10.7)	66.1 (8.3)	<0.001^†^
Height (cm), mean (SD)	162.5 (8.0)	162.5 (8.0)	162.5 (7.6)	0.944^†^
Weight (kg), mean (SD)	63.6 (11.1)	63.9 (11.0)	61.7 (11.4)	0.036^†^
BMI, mean (SD)	24.0 (3.2)	24.1 (3.2)	23.3 (3.4)	0.007^†^
Smoking pack-years, mean (SD)	15.7 (24.7)	11.6 (21.2)	36.4 (30.2)	<0.001^†^
Biomass fuel exposure, n (%)	118 (14.6)	84 (12.5)	34 (25.4)	<0.001*
Long-term exposure to dust or chemical particles, *n* (%)	182 (22.6)	128 (19.0)	54 (40.3)	<0.001*
Allergy history, *n* (%)	57 (7.1)	42 (6.2)	15 (11.2)	0.064*
Family history of respiratory diseases, *n* (%)	236 (29.3)	195 (29.0)	41 (30.6)	0.793*
Childhood Respiratory Disease, *n* (%)	103 (12.8)	74 (11.0)	29 (21.6)	0.001*
LFQ	18.08 (3.77)	19.05 (3.00)	13.22 (3.49)	<0.001^†^
Modified-IPAG	18.60 (7.90)	16.79 (6.78)	27.69 (6.79)	<0.001^†^
COPD-PS	3.50 (2.31)	2.96 (1.97)	6.19 (2.01)	<0.001^†^
IPAG	18.64 (6.37)	17.35 (5.72)	25.13 (5.43)	<0.001^†^
COPD-SQ	10.80 (5.09)	9.83 (4.69)	15.66 (4.15)	<0.001^†^
SCSQ	2.67 (2.43)	2.17 (2.08)	5.16 (2.50)	<0.001^†^

*Pearson χ^2^ test;

†Independent *t* test; SD, standard deviation; BMI, body mass index; COPD, chronic obstructive pulmonary disease; LFQ, lung function questionnaire; COPD-PS, COPD population screener questionnaire; IPAG, international primary care airways group questionnaire; COPD-SQ, COPD screening questionnaire; SCSQ, the salzburg COPD screening questionnaire.

### 3.2 Diagnostic performance of COPD screening questionnaires

At recommended cut-off values (Table 2), the AUC for the six questionnaires ranged from 0.681 (SCSQ) to 0.796 (COPD-PS). Modified-IPAG showed the highest sensitivity (94.78%) and NPV (98.11%), while COPD-SQ demonstrated the highest specificity (87.05%) and PPV (43.87%). Pairwise comparison of AUCs using the DeLong test with Bonferroni correction indicated that COPD-PS had significantly higher AUC than IPAG (*P* < 0.001), COPD-SQ (*P* < 0.001), and SCSQ (*P* < 0.001). In contrast, no statistically significant differences were observed between COPD-PS and LFQ (*P* = 1.000), COPD-PS and Modified-IPAG (*P* = 0.130), Modified-IPAG and LFQ (*P* = 1.000), Modified-IPAG and COPD-SQ (*P* = 0.330), IPAG and COPD-SQ (*P* = 1.000), IPAG and SCSQ (*P* = 1.000), or COPD-SQ and SCSQ (*P* = 1.000) (Table 3).

**TABLE 2:** Diagnostic performance of COPD screening questionnaires.

Questionnaire	Cut-off	Sen (%)	Spe (%)	PPV (%)	NPV (%)	ACC (%)	AUC
LFQ	≤18	91.79	62.50	32.80	97.45	67.37	0.772
Modified- IPAG	≥17	94.78	54.02	29.13	98.11	60.79	0.744
COPD-PS	≥5	79.85	79.32	43.50	95.18	79.40	0.796
IPAG	≥17	91.79	47.92	26.00	96.70	55.21	0.699
COPD-SQ	≥16	50.75	87.05	43.87	89.86	81.02	0.689
SCSQ	≥2	89.55	46.73	25.10	95.73	53.85	0.681

Sen, sensitivity; Spe, specificity; PPV, positive predictive value; NPV, negative predictive value; ACC, accuracy; AUC, area under the curve.

**TABLE 3:** Pairwise comparison of AUCs (*Adjusted *P* values).

Questionnaire	LFQ	Modified- IPAG	COPD-PS	IPAG	COPD-SQ	SCSQ
LFQ	–	–	–	–	–	–
Modified- IPAG	1.0	–	–	–	–	–
COPD-PS	1.0	0.130	–	–	–	–
IPAG	0.001	0.009	<0.001	–	–	–
COPD-SQ	0.009	0.330	<0.001	1.0	–	–
SCSQ	<0.001	0.011	<0.001	1.0	1.0	–

*Adjusted *P* values from DeLong test with Bonferroni correction (*P* < 0.05 indicates statistical significance).

### 3.3 Dynamic changes in screening accuracy across prevalence levels

**FIGURE 1:**
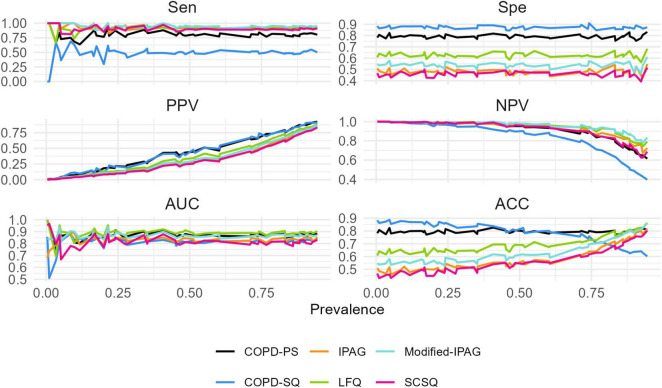
Screening performance of six COPD questionnaires across prevalence levels.

This study simulated the screening ACC of six questionnaires across a prevalence spectrum of 5%–95% (Figure 1). Linear regression analysis demonstrated significant positive associations between ACC and prevalence for LFQ (β = 0.200, 95% CI: 0.163–0.237), Modified- IPAG (β = 0.293, 95% CI: 0.258–0.328), IPAG (β = 0.329, 95% CI: 0.301–0.357), and SCSQ (β = 0.313, 95% CI: 0.281–0.345) (all Bonferroni-adjusted *P* < 0.001). A significant negative association was observed for COPD-SQ (β = −0.263, 95% CI: −0.302 to −0.224, *P* < 0.001). No significant association was found for COPD-PS (β = −0.018, 95% CI: −0.041–0.005, *P* = 0.114).

The absolute ACC fluctuation (ΔACC, maximum-minimum difference) ranged from 0.06 (COPD-PS) to 0.35 (IPAG), with values of 0.24 (LFQ), 0.31 (Modified- IPAG), 0.26 (COPD-SQ), and 0.33 (SCSQ). Complete regression statistics are presented in Table 4.

**TABLE 4:** Regression analysis of screening accuracy against prevalence.

Questionnaire	ΔACC	β (Slope)	95% CI for β	*P-value^a^*
LFQ	0.24	0.200	(0.163, 0.237)	<0.001
Modified-IPAG	0.31	0.293	(0.258, 0.328)	<0.001
COPD-PS	0.06	−0.018	(−0.041, 0.005)	0.114
IPAG	0.35	0.329	(0.301, 0.357)	<0.001
COPD-SQ	0.26	−0.263	(−0.302, −0.224)	<0.001
SCSQ	0.33	0.313	(0.281, 0.345)	<0.001

ΔACC, Absolute performance fluctuation, calculated as the difference between maximum and minimum ACC; β, Regression coefficient indicating the change in ACC per unit increase in prevalence;

a, Two-tailed *t*-test was used to assess whether β significantly differed from zero (significance level α = 0.05), with Bonferroni correction for multiple comparisons.

## 4 Discussion

This study conducted a cross-sectional comparison of the static diagnostic performance of six commonly used COPD screening questionnaires (LFQ, Modified-IPAG, COPD-PS, IPAG, COPS-SQ, SCSQ) within a single high-risk cohort (COPD detection rate: 16.63%). Innovatively, by dynamically simulating a wide range of prevalence rates (5%–95%), it systematically quantified the sensitivity of each questionnaire’s screening accuracy to changes in prevalence (i.e., “robustness”). The results revealed distinct response patterns in the performance of different COPD screening questionnaires to dynamic changes in prevalence.

The most striking finding was the exceptional robustness demonstrated by the COPD-PS. In the static assessment, PS showed a balanced diagnostic performance with sensitivity (79.85%) and specificity (79.32%) (AUC 0.796). More crucially, under dynamic simulation across varying prevalence rates, its screening accuracy exhibited minimal fluctuation (ΔACC = 0.06; β = −0.018, *P* = 0.114). This indicates that COPS-PS’s performance remains relatively stable across different prevalence scenarios, largely unaffected by the level of prevalence in the target screening population. This universal robustness likely stems from its balanced sensitivity and specificity (both ≈79%). Furthermore, its items focus on core risk factors (age, smoking history) and core symptoms (breathlessness, productive cough, activity limitation) with globally recognized associations to COPD (1, 34), and it has undergone rigorous cognitive testing to ensure consistent comprehension across diverse populations (25).

In stark contrast to COPS-PS, other questionnaires exhibited significant “scenario dependency.” Although the COPD-SQ questionnaire achieved the highest specificity (87.05%) and the lowest sensitivity (50.75%) in the static assessment (a pattern maintained in the dynamic evaluation), its accuracy showed a sharp decline as prevalence increased (β = −0.263, *P* < 0.001), with a substantial fluctuation magnitude of 26% (ΔACC = 0.26). This makes COPD-SQ the optimal tool for low-prevalence scenarios. When the estimated prevalence is below 30%, its high specificity effectively controls false positives and unnecessary referrals while maintaining the highest accuracy (ACC > 80%). However, its accuracy significantly decreases in high-prevalence scenarios (>50%). Conversely, the Modified-IPAG and LFQ questionnaires, leveraging their ultra-high sensitivity (94.78% and 91.79%, respectively) and NPV (98.11% and 97.45%, respectively), performed optimally for rule-out screening, minimizing missed diagnoses (false negatives). It is important to note, however, that their accuracy improves with increasing prevalence (Modified-IPAG β = 0.293, LFQ β = 0.200, both *P* < 0.001), and their fluctuation range is also larger (ΔACC = 0.31 and 0.24, respectively). This implies that in very low prevalence scenarios, their relatively lower specificity may lead to an increased false positive rate. The IPAG and SCSQ performed relatively poorly in both this study and the dynamic simulations (lower AUC, poor robustness), highlighting inherent limitations of the tools or insufficient validation.

**FIGURE 2:**
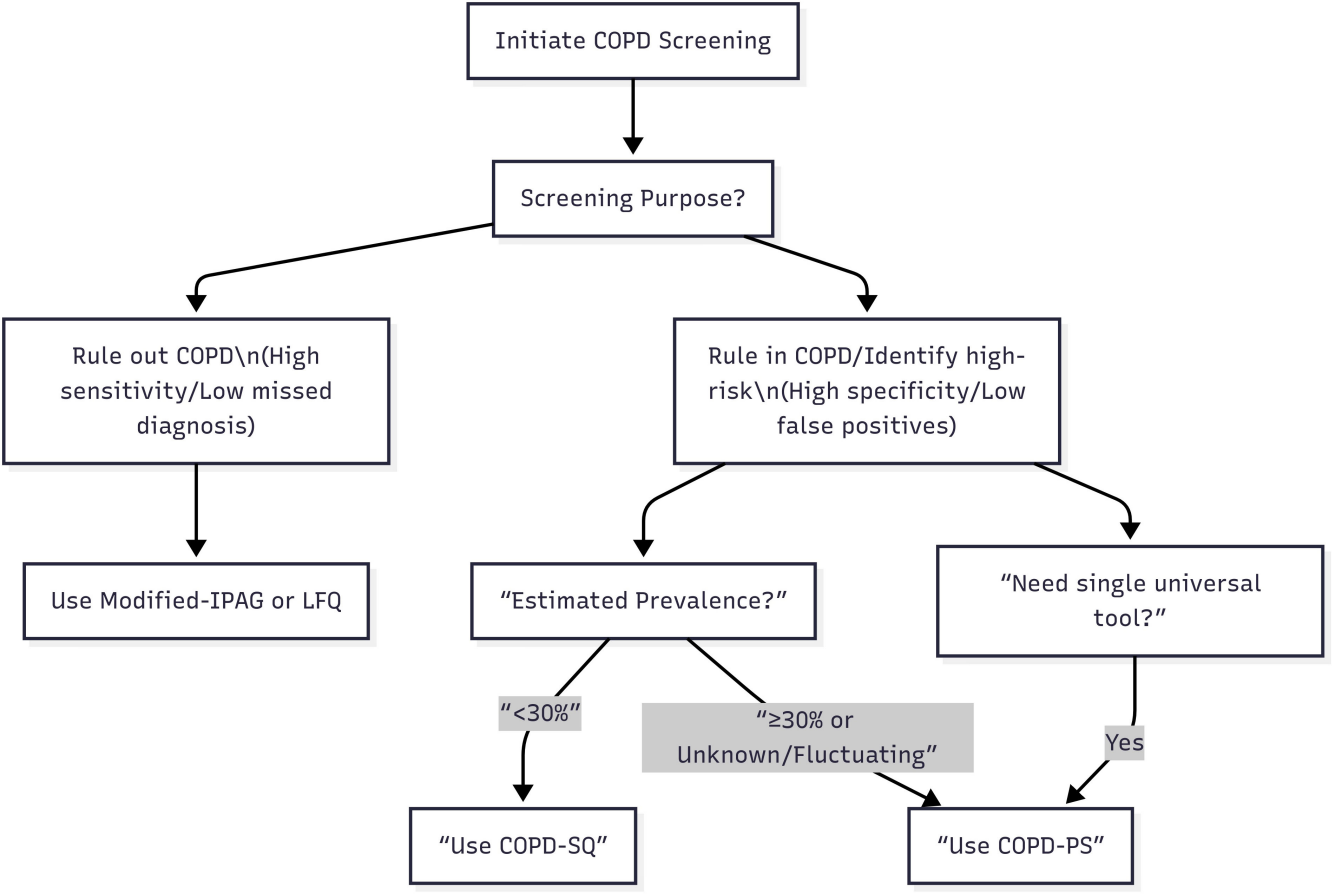
Adaptive COPD screening protocol based on purpose and population prevalence.

Based on a comprehensive analysis of the static performance characteristics and dynamic robustness of the six screening questionnaires, this study proposes a “Scenario-First” adaptive screening strategy for COPD. Specifically, when the core objective is “ruling out COPD” (prioritizing minimizing missed diagnosis risk), such as during large-scale initial screening in primary care or in settings with extremely limited referral resources needing rapid identification of individuals highly unlikely to have COPD to conserve advanced diagnostics, the Modified-IPAG or LFQ questionnaires should be prioritized; leveraging their ultra-high sensitivity (both >90%) and NPV (both >97%), these efficiently identify non-COPD individuals, minimizing missed diagnoses, though this may result in a higher false positive rate manageable through subsequent spirometry. Conversely, when the core objective is “ruling in COPD or identifying high-risk populations” (prioritizing referral accuracy and controlling false positives), such as during pre-referral screening in specialist clinics or screening high-exposure groups (e.g., smokers, occupational dust), the choice must consider estimated prevalence: if prevalence is low (<30%), the COPD-SQ questionnaire is optimal due to its outstanding high specificity (>87%), effectively reducing false positives and unnecessary referrals; if prevalence is high (≥30%), difficult to determine, likely to fluctuate significantly (e.g., respiratory clinic patients, high-exposure occupations), or a more stable tool is needed, the COPD-PS questionnaire should be prioritized, as it maintains relatively good specificity and PPV at high prevalence (outperforming COPD-SQ’s sharp decline) and its exceptional robustness makes it the “safe choice” and versatile first-line tool for uncertain or variable contexts. Furthermore, for institutions needing a single, universal tool to adapt to diverse scenarios or simplify processes, COPD-PS is undoubtedly the most reliable and adaptable choice due to its exceptional robustness. This strategy transcends the limitations of traditional comparisons based solely on single, fixed prevalence scenarios. Its core principle lies in precisely matching the optimal screening tool to the specific screening objective and the epidemiological background of the target population (primarily estimated prevalence), providing actionable, evidence-based decision-making support for clinical and public health practice (Figure 2).

## 5 Limitations

However, this study has certain limitations. Firstly, the prevalence simulation assumed a linear effect, whereas real-world COPD prevalence may exhibit non-linear patterns. Secondly, the study is based on single-center data; future external validation in multi-center cohorts is warranted. Finally, the cultural adaptation of questionnaire items and participants’ comprehension ability may also impact performance, a common challenge for all self-administered screening tools. Future research could explore integrating risk prediction models with questionnaire results to develop more intelligent dynamic decision support tools and evaluate the cost-effectiveness and implementation feasibility of this strategy in real-world application settings.

## 6 Conclusion

By dynamically simulating prevalence rates, this study quantitatively revealed a key reason for the heterogeneity observed in the diagnostic performance of COPD screening questionnaires across previous studies: the significant influence of target population prevalence on tool performance. Based on this insight, we innovatively propose a “Scenario-First” screening strategy. This strategy dynamically matches the optimal tool based on estimated prevalence and the screening objective, thereby transcending the limitations of the traditional “tool-first” static framework and providing an evidence-based decision-making foundation for addressing heterogeneity in screening scenarios.

## Data Availability

The original contributions presented in this study are included in this article/supplementary material, further inquiries can be directed to the corresponding author/s.
